# Transcrystalline growth of PLLA on carbon fiber grafted with nano-SiO_2_ towards boosting interfacial bonding in bone scaffold

**DOI:** 10.1186/s40824-021-00248-0

**Published:** 2022-01-20

**Authors:** Pei Feng, Jiye Jia, Shuping Peng, Yang Shuai, Hao Pan, Xinna Bai, Cijun Shuai

**Affiliations:** 1 State Key Laboratory of High Performance Complex Manufacturing, College of Mechanical and Electrical Engineering, Central South University, Changsha, 410083 China; 2 NHC Key Laboratory of Carcinogenesis of Hunan Cancer Hospital and the Affiliated Cancer Hospital of Xiangya School of Medicine, Cancer Research Institute, School of Basic Medical Science, Central South University, Changsha, 410013 Hunan China; 3 The Key Laboratory of Carcinogenesis and Cancer Invasion of the Chinese Ministry of Education, Xiangya Hospital, Central South University, Changsha, 410078 Hunan China; 4 College of Life Science and Technology, Huazhong University of Science and Technology, Wuhan, 430074 China; 5 Department of Periodontics & Oral Mucosal Section, Xiangya Stomatological Hospital & Xiangya School of Stomatology, Central South University, Changsha, 410013 China; 6 Department of Conservative Dentistry & Endodontics, Xiangya Stomatological Hospital & Xiangya School of Stomatology, Central South University, Changsha, 410013 China; 7 Institute of Additive Manufacturing, Jiangxi University of Science and Technology, Nanchang, 330013 China

**Keywords:** Carbon fiber, Poly (lactic acid), Bone scaffold, Interfacial crystallization

## Abstract

**Background:**

The reinforcement effect of fiber-reinforced polymer composites is usually limited because of the poor interfacial interaction between fiber and polymer, though fiber reinforcement is regarded as an effective method to enhance the mechanical properties of polymer.

**Methods:**

In this study, nano-SiO_2_ particles grafted by 3-Glycidoxypropyltrimethoxysilane (KH560) were introduced onto the surface of 3-Aminopropyltriethoxysilane (KH550) modified carbon fiber (CF) by a self-assembly strategy to improve the interfacial bonding between CF and biopolymer poly (lactic acid) (PLLA).

**Results:**

The results indicated that PLLA chains preferred to anchor at the surface of nano-SiO_2_ particles and then formed high order crystalline structures. Subsequently, PLLA spherulites could epitaxially grow on the surface of functionalized CF, forming a transcrystalline structure at the CF/PLLA interface. Meanwhile, the nano-SiO_2_ particles were fixed in the transcrystalline structure, which induced a stronger mechanical locking effect between CF and PLLA matrix. The results of tensile experiments indicated that the PLLA/CF-SiO_2_ scaffold with a ratio of CF to SiO_2_ of 9:3 possessed the optimal strength and modulus of 10.11 MPa and 1.18 GPa, respectively. In addition, in vitro tests including cell adhesion and fluorescence indicated that the scaffold had no toxicity and could provide a suitable microenvironment for the growth and proliferation of cell.

**Conclusion:**

In short, the PLLA/CF-SiO_2_ scaffold with good mechanical properties and cytocompatibility had great potential in the application of bone tissue engineering.

## Introduction

Fiber-reinforced technique is regarded as an effective method to enhance the mechanical properties of polymers because of the many advantages of fiber materials such as high strength, modulus and stiffness [1, 2]. Among various fibers, carbon fiber (CF) with high strength and modulus which can reach 942 MPa and 42.8 GPa, respectively, is considered as the most promising fiber material acting as reinforcement phase [3, 4]. The reinforcement effect of CF can be achieved by means of that CF bears the loading transferred from polymer matrix and absorbs partial fracture energy. For this purpose, good interfacial bonding between CF and polymer is necessary because it can ensure efficient loading transfer and reduce stress concentration [5–7]. However, the interfacial interaction between CF and polymer is weak on account of the smooth surface and low activity of CF, which limits the reinforcement efficiency of CF [8, 9].

Generally, some methods including increasing surface roughness and introducing activity groups have been reported to improve the interfacial bonding between CF and polymer. Yang et al. [10] studied the effect of CF treated by sandpaper rubbing on the interfacial adhesive strength, and the results indicated that the tensile strength of CF reinforced composite increased with the decrease of the grit size of sandpaper, which was mainly attributed to that the increased roughness formed stronger mechanical locking. Lin et al. [11] used nitrogen and air as plasma gases to treat CF and evaluated the effect of treated CF on the mechanical properties of polymer. The results indicated that some polar chemical functional groups (−NH_2_ and -OH) were generated after treating by plasma gases, which enhanced the interfacial strength between CF and polymer. Although these methods can enhance the interfacial interaction between CF and polymer to a certain extent, mechanical defects will be introduced on the surface of CF, which weakens the reinforcement effect [12].

Recently, introducing nanomaterials onto the surface of CF is an interesting way to improve the interfacial properties of CF-reinforced polymers [13–15]. On one hand, the nanomaterials anchored on CF surface can increase the surface roughness, which is beneficial to form stronger mechanical locking force at the interface. On the other hand, nanomaterials are generally regarded as good nucleating agents for the crystallization of polymer. When nanomaterials are introduced on the surface of CF, it is possible to construct an interfacial crystallization structure between CF and polymer [16, 17]. Nano-SiO_2_ as a kind of nanomaterial possesses a lot of excellent properties including large specific surface area and high surface activity [18]. This is beneficial to enhance the interfacial bonding between CF and polymer by forming mechanical locking and interfacial crystallization structure. And many studies have been reported that nano-SiO_2_ can be utilized in different applications of biomedicine due to its non-toxicity and biological activity [19, 20].

Herein, nano-SiO_2_ was grafted on the surface of CF (CF-SiO_2_) to enhance the interfacial bonding between CF and polymer in bone scaffold fabricated by selective laser sintering (SLS). Poly (lactic acid) (PLLA) was used as the scaffold material for bone tissue engineering due to its good biodegradability and biocompatibility [21–23]. Fourier transform infrared (FTIR) and X-ray photoelectron spectroscopy (XPS) were applied to characterize the chemical structures and functional groups. Scanning electron microscope (SEM) was used to observe the surface morphology of CF before and after grafting with nano-SiO_2_. Polarized optical microscopy (POM) was performed to investigate the crystallization morphology of PLLA at the presence of CF-SiO_2_. The tensile tests were used to evaluate the tensile properties including strength and modulus of PLLA/CF-SiO_2_ scaffolds, and the reinforcement mechanism was analyzed in detail. In addition, cell adhesion and fluorescence tests were performed to investigate the cytocompatibility of the scaffolds.

## Materials and methods

### Materials

CF with carboxyl groups (CF-COOH) was purchased from Cangzhou Zhongli New Material Technology Co., Ltd. (Hebei, China), and the average diameter and aspect ratio of CF were 7 μm and 10 ~ 15, respectively. SiO_2_ nanoparticles with hydroxyl groups (SiO_2_-OH) were purchased from Beijing Deke Daojin Science and Technology Co., Ltd. (Beijing, China), and the average size of SiO_2_ was 30 nm. PLLA powder with a 150 μm average diameter was obtained from Shenzhen PolymtekBiomaterial Co., Ltd. (Shenzhen, China). The coupling agents 3-Aminopropyltriethoxysilane (KH550) and 3-Glycidoxypropyltrimethoxysilane (KH560) were purchased from Aladdin Reagents Co., Ltd. (Shanghai, China). Phosphate buffer saline (PBS), toluene and ethanol were purchased from Sinopharm Chemical Reagent Co., Ltd. (Beijing, China). Fetal bovine serum was purchased from Cellgro Mediatech Inc. (USA). Paraformaldehyde, calcein-AM and propidium iodide were provided by SigmaAldrich CO. (USA).

### Preparation of CF-SiO_2_ hybrid

**Fig. 1:**
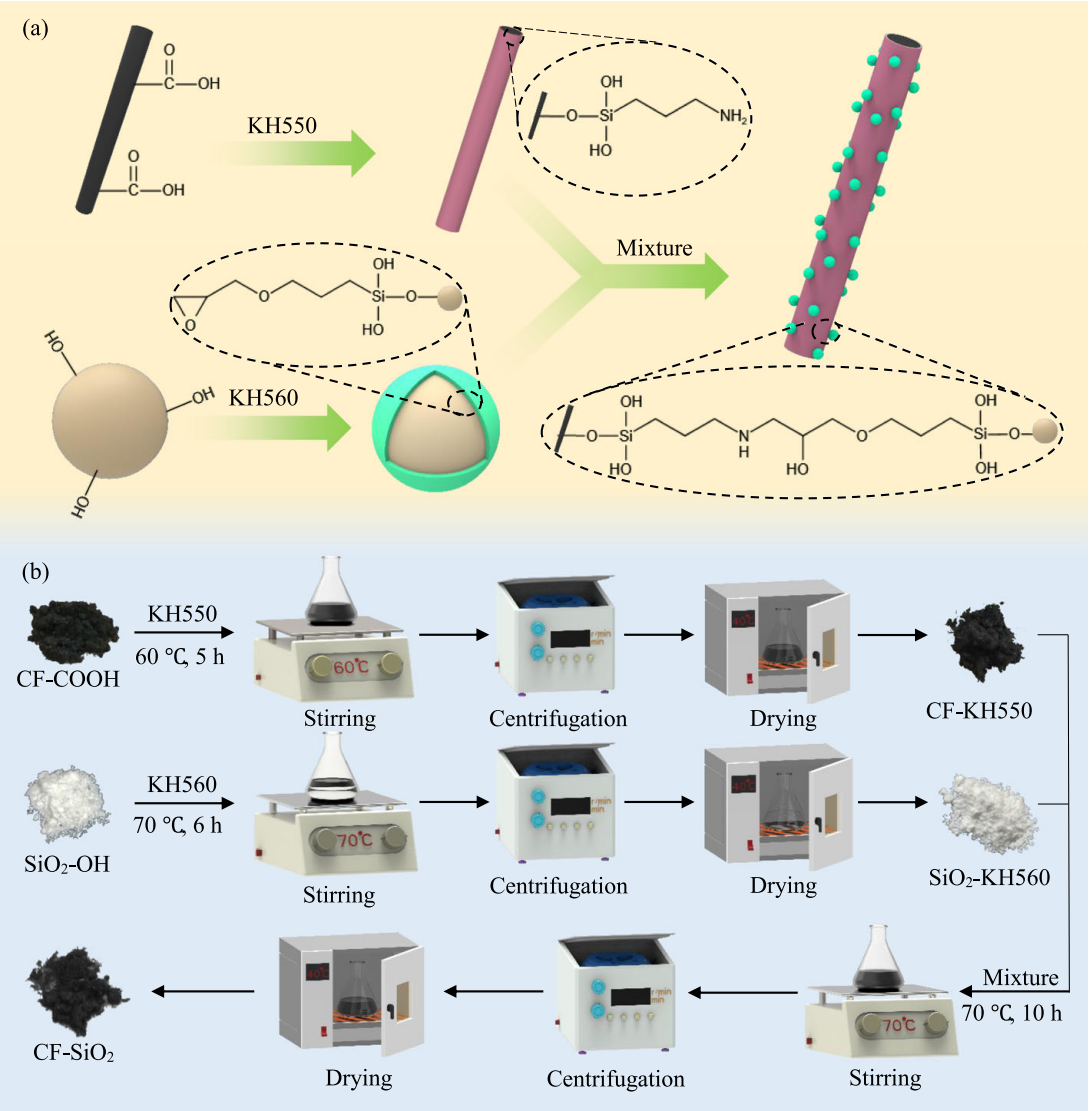
(**a**) Schematic of fabricating CF-SiO_2_ hybrid via a self-assembly strategy. (**b**) Schematic diagram of CF-SiO_2_ hybrid fabrication process

The preparation processes of the CF-SiO_2_ hybrid are presented in Fig. 1. According to the study of Lan et al. [24], KH550 could be introduced to the surface of CF via the reaction of hydroxyl groups and silanol groups. Briefly, 2.0 g of CF-COOH was added in 50 mL of ethanol solution and ultrasonically dispersed for 5 min, and then 1 mL of KH550 was added at room temperature. The mixed solution was reacted at 60 °C for 5 h under constant stirring. After that, CF-KH550 was obtained by centrifuging the mixed solution, washing for three times with ethanol and drying in a vacuum oven. Zhang et al. [25] reported a way of grafting KH560 onto the surface of nano-SiO_2_. Briefly, 2.0 g of SiO_2_ was suspended in 50 mL of toluene solution and ultrasonically dispersed for 30 min, and then 1 mL of KH560 was added drop by drop to the SiO_2_ suspension. Subsequently, the mixed solution was constantly stirred at 70 °C for 6 h. And SiO_2_-KH560 was obtained by centrifuging the mixed solution, washing repeatedly with ethanol, drying in a vacuum oven. A certain amount of the CF-KH550 was added to the SiO_2_-KH560 solution, and the mixed solution was stirred at 70 °C for 6 h. The CF-SiO_2_ hybrid was obtained by centrifuging and drying in a vacuum oven. The weight ratio of the CF-KH550 to the SiO_2_-KH560 was 9:1, 9:3 and 9:5, and they were marked as CF-SiO_2_–1, CF-SiO_2_–2, and CF-SiO_2_–3, respectively.

### Characterizations of CF-SiO_2_ hybrid

The functional groups on the surface of CF-COOH before and after modification were obtained in a FTIR spectrometer (Massachusetts, USA). All specimens were prepared in powder, and the tests were performed in a wavenumber range of 500 to 4500 cm^− 1^ with a resolution of 4 cm^− 1^. XPS (Thermo Scientific K-Alpha) tests were carried out to detect the elemental compositions of specimens using an Al Kα (1486.5 eV) X-ray source at a base pressure of 5 × 10^− 7^ mbar. SEM (Shanghai, China) was used to observe the surface morphologies of CF-COOH before and after modification, and energy dispersive spectroscopy (EDS) (Shanghai, China) was applied to analyze the distribution of C, O, N and Si elements. All the specimens were coated with a thin layer of gold before SEM test to obtain clear images.

### Scaffold fabrication

The scaffold used for tensile and cytocompatibility tests was fabricated by a SLS system [26–30]. PLLA and CF-SiO_2_–1 powder in a ratio of 95 to 5 (PLLA/CF-SiO_2_–1, w/w) were mixed in a beaker with 30 mL ethanol, and the solution was mixed by ultrasonic and constant stirring. Then, the composite powder was obtained by filtering and drying the mixed solution at 50 °C for 24 h. Repeating the process, PLLA/CF, PLLA/CF-SiO_2_–2 and PLLA/CF-SiO_2_–3 composite powders were obtained. The laser sintered the composite powder selectively according to the shape. After that, the sintering platform moved down a height, and the powder delivery system raised the same height to spread a layer of powder on the platform. The height was the same as the thickness of the layer, and the value was approximately 0.2 mm. Repeating the process, the scaffold was fabricated successfully. During the process, the laser power was 2.2 W, and the scanning rate was 120 mm/s.

### Crystallization morphologies

POM (Shanghai, China) equipped with Batuo a BT-3000 hot stage was used to observe the crystallization process of the PLLA, PLLA/CF and PLLA/CF-SiO_2_. Specimens were heated to 200 °C and maintained for 3 min to eliminate the thermal history on a hot stage. Then, the temperature of the hot stage was cooled to 135 °C at a rate of 30 °C/min, and the isothermal crystallization of all specimens proceeded for 3, 6, 9 and 12 min. The crystallization process of specimens was observed, and the crystal morphologies were obtained by a camera. In order to reveal the influence of the grafted nano-SiO_2_ on PLLA crystallization, SEM was also applied to observe the crystallization morphologies of PLLA after keeping it for 20 min at 135 °C. The morphologies of crystallized specimens were examined at an accelerating voltage of 15 kV.

### Mechanical properties

Tensile tests were carried out according to standard ISO 37–2005 [31, 32]. The scaffold specimens were fabricated by SLS. And the shape of specimens for tensile test was dumbbell-shaped (12 × 2 × 2 mm^3^). The tensile properties were assessed by a mechanical testing machine (Shandong, China) at room temperature. In the tensile test, the crosshead speed was 0.5 mm/min. Five scaffold specimens were measured in every group to obtain the average value and standard deviation. After the tensile test, the fracture morphologies of scaffolds were observed by SEM to examine the interface between PLLA and CF.

### Cytocompatibility

Human osteoblast-like cells (MG63 cells, American Type Culture Collection, Rockville, USA) were selected and cultured for 1, 3 and 5 d on scaffold specimens (10 × 10 × 4 mm^3^) to evaluate the cytocompatibility by cell adhesion and fluorescence tests. Specifically, the specimens before cell seeding were sterilized by ultraviolet radiation for 30 min. Then MG63 cells were incubated at 37 °C in Dulbecco’s modified Eagle’s medium (DMEM) containing 10% fetal bovine serum under a humidified 5% CO_2_ atmosphere. After that, the cells were seeded on the surface of each specimen at a concentration of 5 × 10^4^ cell/cm^2^ and cultured in DMEM for 1, 3 and 5 d. When the cell culture was finished, the specimens with the cells were fixed with 2.5% glutaraldehyde and washed with PBS. The morphologies of MG63 cells were observed by SEM after the specimens were dried. And the data of cell relative area was counted from cell adhesion results. The fluorescence analysis was applied to evaluate the cell viability after culturing for 1, 3 and 5 d. Then the cells on the surface of specimens were rinsed by PBS, and the cells were stained by culturing in solution with calcein-AM and propidium iodide for 40 min. The morphologies of the stained cell were observed by a fluorescence microscope (TE2000-S; Nikon), and the living cells were represented by green in the stained images. And the data of cell density was counted from fluorescence results.

### Statistical analysis

The statistical significance of the experiment results was evaluated by SPSS (IBM Co., USA) software, and the obtained data were presented as mean ± standard deviation. The differences with P<0.05 (*) were considered significant, and the differences with P<0.01 (**) were considered highly significant.

## Results

### Preparation of CF-SiO_2_ hybrid

Figure 1 shows the schematic and process of fabricating CF-SiO_2_ hybrid. KH550 was grafted on the surface of CF by the reaction of -COOH on CF and -OH in KH550. KH560 was grafted on the surface of nano-SiO_2_ particles by the reaction of -OH on nano-SiO_2_ particles and -OH in KH560. The CF-SiO_2_ hybrid was fabricated by the reaction of -NH_2_ in CF-KH550 and epoxy group in SiO_2_-KH560.

### Characterization of CF-SiO_2_ hybrid

**Fig. 2:**
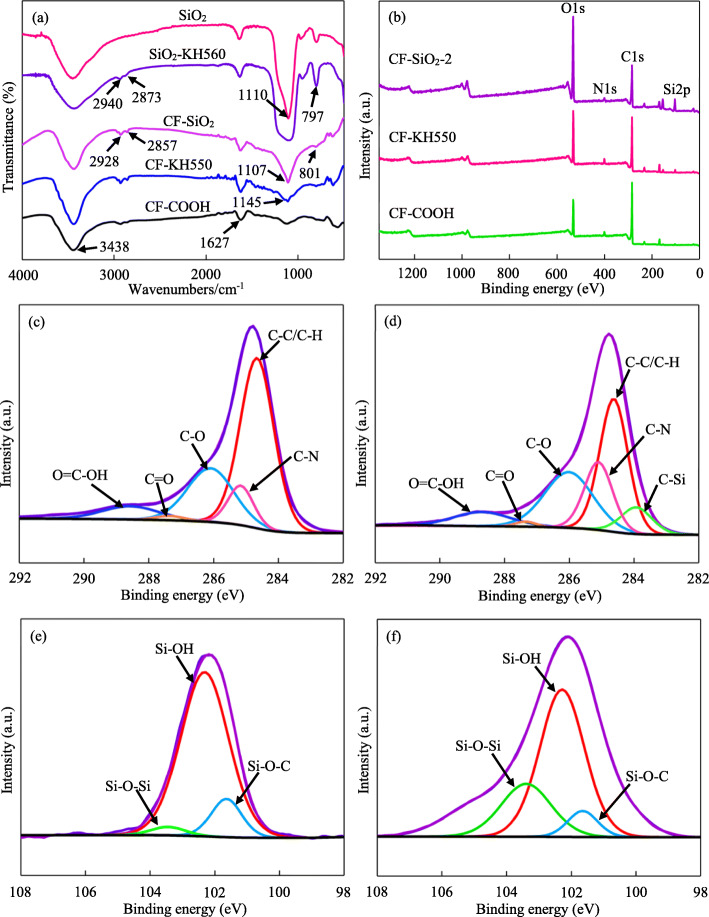
(**a**) FTIR spectra of nano-SiO_2_ and CF before and after modification by KH560 and KH550, respectively. (**b**) Wide-scan survey XPS spectra of CF, CF-KH550 and CF-SiO_2_–2. XPS curve fitting of the C1s peak of CF (**c**) and CF-KH550 (**d**) and the Si2p peak of CF-KH550 (**e**) and CF-SiO_2_–2 (**f**)

Figure 2 a shows the FTIR spectra of nano-SiO_2_ and CF before and after modification by KH560 and KH550, respectively. In the spectrum of SiO_2_, there was no peak and the curve was smooth from 2750 cm^− 1^ to 3000 cm^− 1^. In the spectrum of SiO_2_-KH560, two absorption peaks at 2940 cm^− 1^ and 2873 cm^− 1^ attributable to the -CH_3_ and -CH_2_- stretching vibration in KH560, respectively, were observed [33, 34]. For the CF-COOH specimen, the obvious absorption peak of -OH at 3438 cm^− 1^ and stretch vibration peak of -C=O at 1627 cm^− 1^ were observed. After modification by KH550, the peaks of the -CH_3_ group and -CH_2_- group at 2928 cm^− 1^ and 2857 cm^− 1^ were more evident in the CF-KH550 spectrum. Moreover, the peak at 1145 cm^− 1^ which was ascribed to the bending vibration of C-O-Si was found. In the CF-SiO_2_ spectrum, the peak at 1107 cm^− 1^ ascribing to the antisymmetric vibration of the Si-O-Si group emerged, and there appeared a peak at 801 cm^− 1^ which was attributed to the symmetrical stretching vibrations of the Si-O group [35].

XPS, as an effective technique, is applied to analyze the surface elements and functional groups [36]. Figure 2 b shows the wide-scan survey XPS spectra of CF, CF-KH550 and CF-SiO_2_–2. Table 1 shows the content of C, O, N and Si elements in CF, CF-KH550 and CF-SiO_2_–2 specimens obtained from XPS results. It could be observed that there were distinct C and O peaks in CF-COOH, and the content of the C element was higher than that of the O element. After modified by the coupling agent KH550, the relative content of N and Si elements increased to 4.47 and 3.39%, respectively. In the CF-SiO_2_–2 spectrum, the content of the O element increased from 28.53 to 40.67%, and the content of Si element increased from 3.39 to 13.60%. To further evaluate the chemical bonding between CF and nano-SiO_2_, the C1s peaks of CF-COOH and CF-KH550 and the Si2p peaks of CF-KH550 and CF-SiO_2_–2 were obtained from the high-resolution XPS spectra by peak fitting (Figs. 2 c-f). For the CF-COOH specimen, the C1s peak was deconvoluted into five binding energies at 284.7 eV (C-C), 285.2 eV (C-N), 286.1 eV (C-O), 287.4 eV (C=O) and 288.7 eV (O=C-OH) (Fig. 2 c) [37, 38]. After modification by KH550, there emerged a new binding energy C-Si peak in CF-KH550 specimen, and the C-N peak was enhanced significantly when compared with that of CF-COOH specimen (Fig. 2 d). In addition, the Si2p peaks of CF-KH550 and CF-SiO_2_–2 were fitted to three binding energy peaks at 102.3 eV, 103.4 eV and 101.7 eV, which were assigned to Si-OH, Si-O-Si and Si-O-C, respectively [39, 40], as shown in Figs. 2 e-f.

**Table 1:** The content of C, O, N and Si elements in CF, CF-KH550 and CF-SiO_2_–2 specimens obtained from XPS results

Specimens	Element content (%)			
	C1s	O1a	N1a	Si2p
CF	73.11	21.80	3.77	1.32
CF-KH550	63.62	28.53	4.47	3.39
CF-SiO_2_–2	43.04	40.67	2.68	13.60

### Surface morphologies analysis

**Fig. 3:**
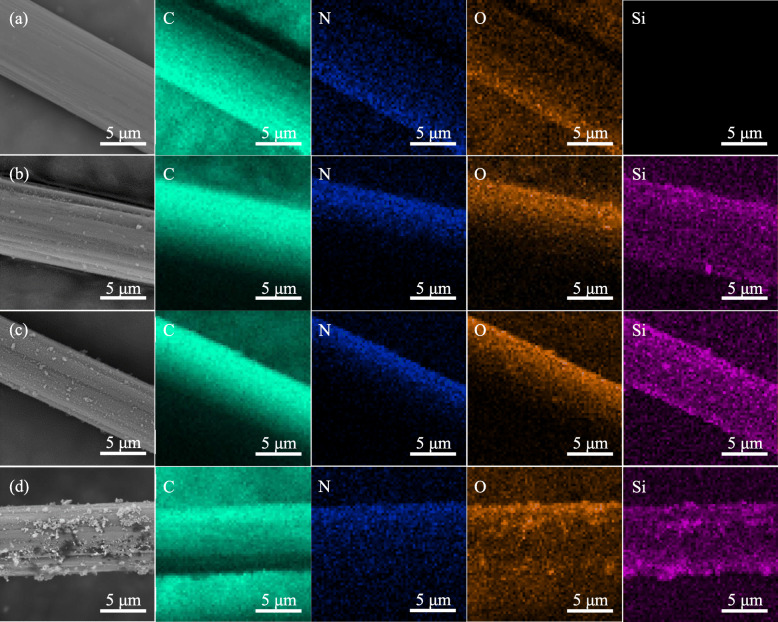
SEM and EDS images of different specimens surface: (**a**) CF, (**b**) CF-SiO_2_–1, (**c**) CF-SiO_2_–2 and (**d**) CF-SiO_2_–3. C, N, O and Si represent the element mapping of carbon, nitrogen, oxygen and silicon elements, respectively

Figure 3 shows the SEM and EDS images of different specimens surface: CF, CF-SiO_2_–1, CF-SiO_2_–2 and CF-SiO_2_–3. It could be observed that the difference of surface morphologies of CF modified by nano-SiO_2_ with different content was apparent. For the CF specimen, there was a smooth surface on CF and no Si element was observed (Fig. 3 a). After modification by nano-SiO_2_ of low concentration, there appeared some nano-SiO_2_ particles, and they distributed uniformly (Fig. 3 b). When the content of nano-SiO_2_ increased, there appeared more nano-SiO_2_ particles which were uniformly distributed on the surface of CF (Fig. 3 c). But for the CF-SiO_2_–3 specimen, lots of agglomerates were observed on CF surface. The EDS results revealed the distribution of nano-SiO_2_ particles on CF surface. For the CF-SiO_2_–1 specimen, Si element with uniform distribution was observed apparently, but the concentration was low. It could be noticed that the Si element in the CF-SiO_2_–2 specimen exhibited the same uniform distribution, and the concentration was higher than that of the CF-SiO_2_–1 specimen. However, it could be obviously observed that Si element showed high concentration locally in the CF-SiO_2_–3 specimen. Compared to the O element of untreated CF, the concentration of O element in the other specimens was higher.

### Crystallization morphologies analysis

**Fig. 4:**
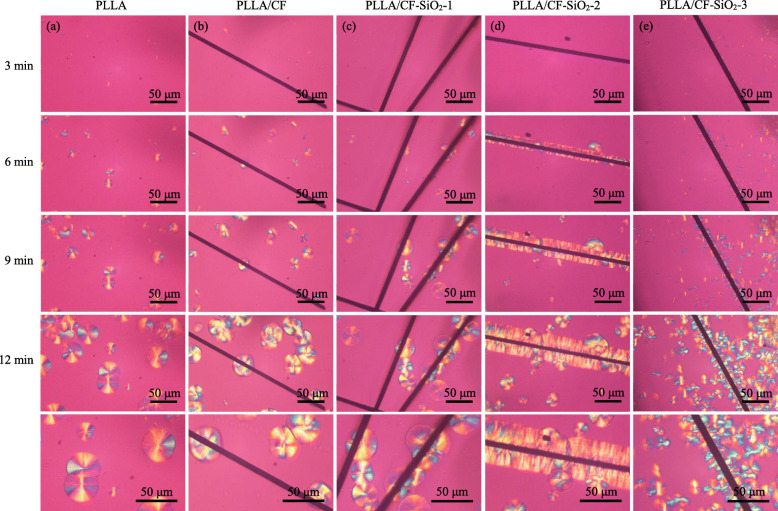
POM images of (**a**) PLLA, (**b**) PLLA/CF, (**c**) CF-SiO_2_–1, (**d**) CF-SiO_2_–2 and (**e**) CF-SiO_2_–3 after holding for 3, 6, 9 and 12 min at 135 °C

Figure 4 shows the POM images of PLLA, PLLA/CF, CF-SiO_2_–1, CF-SiO_2_–2 and CF-SiO_2_–3 after holding for 3, 6, 9 and 12 min at 135 °C. For the PLLA specimen, only some spherulites were observed, and the size of them increased with the extension of holding time (Fig. 4 a). For the PLLA/CF specimen, the spherulites appeared randomly, and there was no crystallization on the surface of CF (Fig. 4 b). However, it was interesting to observe that the spherulites appeared on the surface of CF and grew along with CF in the PLLA/CF-SiO_2_–2 specimen, forming a transcrystalline structure at the interface between CF-SiO_2_–2 and PLLA (Fig. 4 d). Instead, for the PLLA/CF-SiO_2_–3 specimen, many nuclei emerged distinctly along CF surface after holding 3 min at 135 °C, whereas the spherulites grew irregularly with the increase of holding time, which was different from the PLLA/CF-SiO_2_–2 specimen (Fig. 4 e).

**Fig. 5:**
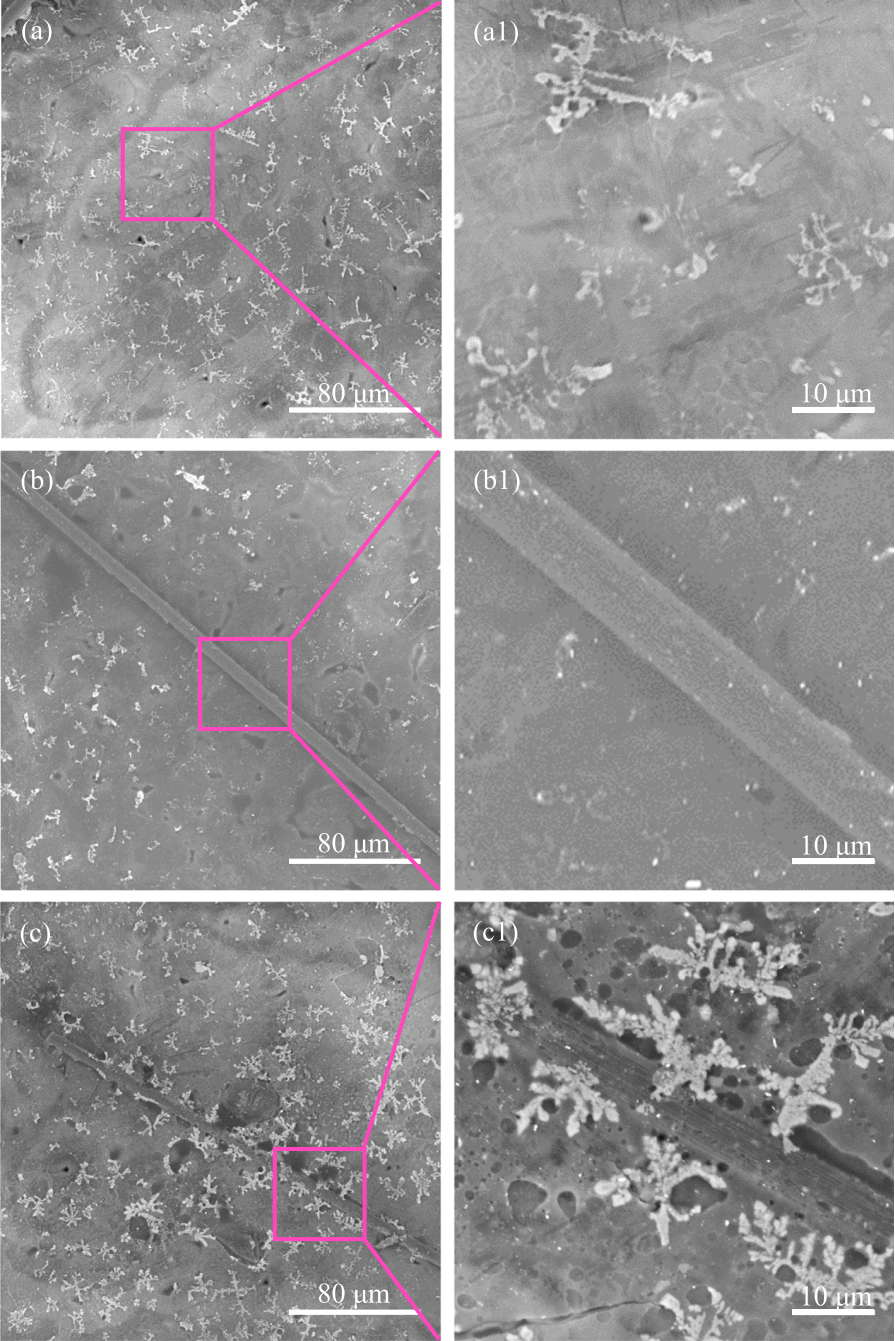
SEM images of (**a-a1**) PLLA, (**b-b1**) PLLA/CF and (**c-c1**) PLLA/CF-SiO_2_–2 specimens after after holding for 12 min at 135 °C. (**a1**), (**b1**) and (**c1**) are the higher magnification images of CF surfaces

Figure 5 shows the SEM images of PLLA, PLLA/CF and PLLA/CF-SiO_2_–2 specimens after after holding for 12 min at 135 °C. Initial crystal morphologies were intuitively observed by SEM to provide insights into the formation of PLLA crystal. It was found that PLLA crystal exhibited branched crystalline morphology at the beginning of crystallization (Figs. 5 a -a1). The same crystal morphology was observed in the PLLA/CF specimen, whereas the surface of CF was still smooth and no crystal appeared. However, it was remarkable that many branched crystalline morphologies appeared on the surface of CF-SiO_2_ in the PLLA/CF-SiO_2_–2 specimen (Figs. 5 c -c1).

### Mechanical properties

**Fig. 6:**
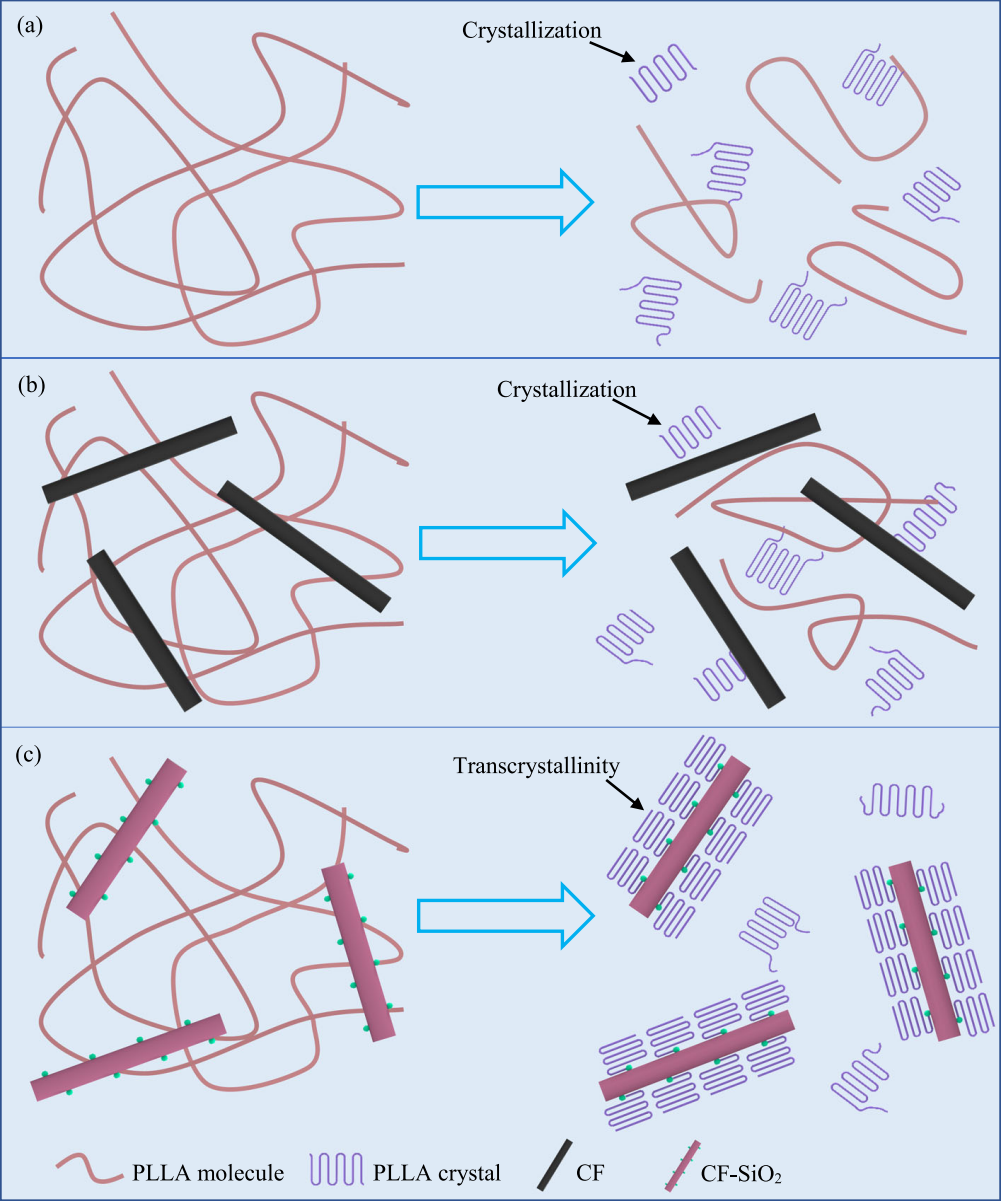
(**a**) The images of tensile specimens with a dumbbell shape. (**b**) The images of specimens before and after tensile tests. (**c**) Tensile stress-strain curves of PLLA, PLLA/CF and PLLA/CF-SiO_2_ specimens. (**d**) Strength and modulus of PLLA, PLLA/CF and PLLA/CF-SiO_2_ specimens. * denotes *p* < 0.05 compared with the PLLA scaffold specimen

Figure 6 shows the images of tensile specimens and the results of tensile tests. The scaffold specimens for tensile tests were fabricated in the shape of a dumbbell by SLS, as shown in Fig. 6 a. And the scaffold images before and after tensile tests are shown in Fig. 6 b. After that, the stress-strain curves, tensile strength and modulus of all specimens were obtained and shown in Fig. 6 c and Fig. 6 d. It could be observed that PLLA specimens possessed the minimum strength and modulus of 7.19 MPa and 0.86 GPa, respectively. The strength and modulus of the specimens were further increased with the addition of CF-SiO_2_. The PLLA/CF-SiO_2_–2 specimen had the maximum strength and modulus of 10.11 MPa and 1.18 GPa, respectively. And the strength and modulus of PLLA/CF-SiO_2_–2 specimen increased by 40.6 and 37.2%, respectively, compared to that of PLLA specimen. However, the strength and modulus of the PLLA/CF-SiO_2_–3 specimen showed a decreasing tendency compared to that of the PLLA/CF-SiO_2_–2 specimen.

**Fig. 7:**
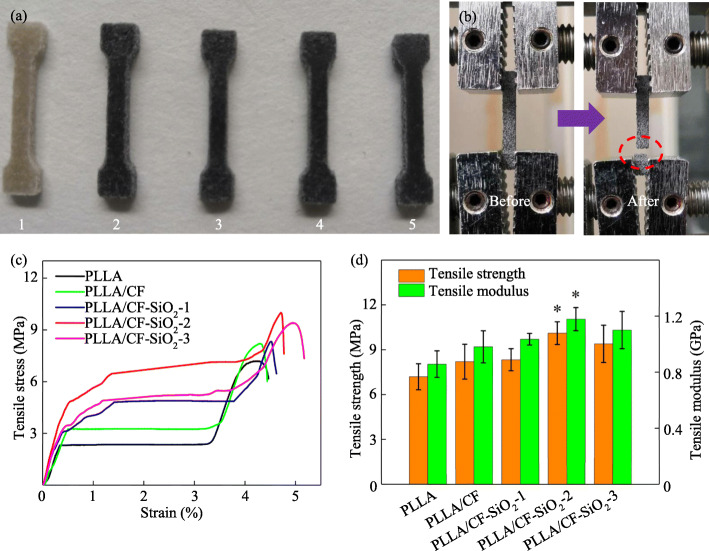
SEM images of fracture surface morphology after tensile tests of (**a**) PLLA, (b) PLLA/CF and (c-d) PLLA/CF-SiO_2_–2. (**a1**) (**b1**) (**c1**) and (**d1**) magnified areas indicate in the orange dotted squares in (**a**) (**b**) (**c**) and (**d**), respectively. (**e**) Schematic diagram of fracture mechanism of PLLA/CF and PLLA/CF-SiO_2_ specimens. Red arrows and circles indicate fiber pulling out and interfacial bonding, respectively

Figure 7 shows the SEM images of fracture surface morphology after tensile tests of PLLA, PLLA/CF and PLLA/CF-SiO_2_–2 specimens. It could be observed that most of the surface of the PLLA specimen was smooth and only a small number of folds appeared (Figs. 7 a -a1). For the PLLA/CF specimen, some holes and pulling-out CF appeared, and it could be observed that there was interspace between CF and PLLA matrix, indicating a weak interfacial bonding between them (Figs. 7 b -b1). For the PLLA/CF-SiO_2_–2 specimen, there were also holes where the fibers were pulled out (Figs. 7 c -c1). However, no interspace between CF-SiO_2_ and PLLA matrix was observed but the matrix was fixed on the surface of CF. The PLLA matrix adhering on the surface of pulling-out CF was observed (Figs. 7 d -d1).

### Cytocompatibility

**Fig. 8:**
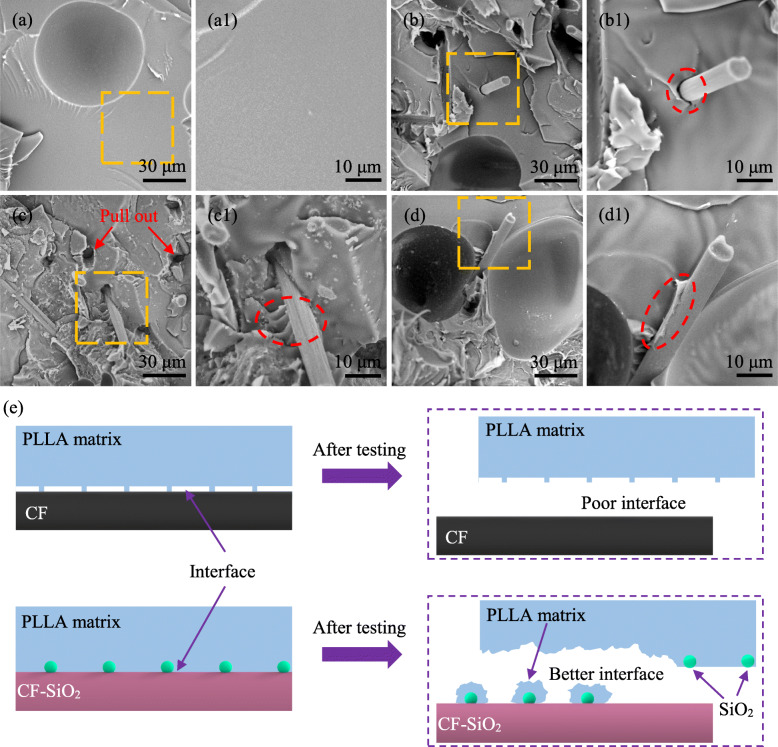
(**a-c**) The fluorescence images of MG63 cells after culturing for 1, 3 and 5 d, respectively. (**d**) The data of cell density counted from fluorescence results. (**e-g**) The SEM images of MG63 cells after culturing for 1, 3 and 5 d. (**h**) The data of cell relative area counted from cell adhesion results. (**a1**), (**b1**), (**c1**), (**e1**), (**f1**) and (**g1**) magnified areas indicate in the orange dotted squares in (**a**), (**b**), (**c**), (**e**), (**f**) and (**g**), respectively. With the extension of incubation time, more filopodia can be observed, and the number and area of MG63 cells increase significantly. * denotes *p* < 0.05 and ** denoted *p* < 0.01 compared with the MG63 cells cultured for 1 d on the PLLA/SiO_2_–2 scaffold specimen

Figure 8 shows the results of in vitro tests including cell adhesion and fluorescence. It could be observed from the results of fluorescence tests (Fig. 8 a-d) that the number of MG63 cells increased significantly with the extension of culture time. Besides, more filopodia were observed on the scaffold cultured for 3 d and 5 d (Fig. 8 b-c). The morphology of MG63 cells cultured on the scaffold for 1, 3 and 5 d, respectively, was observed by SEM, and the results are shown in Fig. 8 e-g. After culturing for 1 d, there appeared many cells with the fusiform and spherical shape on the scaffold (Fig. 8 e -e1). After culturing for 3 d, more filopodia of MG63 cells were observed and many MG63 cells began to fuse (Fig. 8 f -f1). When the incubation time reached 5 d, MG63 cells continued to grow and spread and a cell layer formed. The results of cell relative area also confirmed that MG63 cells covered most of the area of the scaffold, as shown in Fig. 8 h.

## Discussion

To enhance the mechanical properties of CF-loaded composites, the improvement of the interfacial properties between CF and polymer is especially important. Although introducing nano-SiO_2_ onto the surface of CF is an interesting approach to improve the interfacial adhesion between CF and PLLA by constructing nanostructure on the surface of CF, but the content of nano-SiO_2_ on the surface of CF is still one of the critical considerations. From the results of FTIR, the two peaks at 2940 cm^− 1^ and 2873 cm^− 1^ attributable to the -CH_3_ and -CH_2_- stretching vibration in KH560 emerged, which indicated that the surface of nano-SiO_2_ was successfully modified by KH560. And the peaks of the -CH_3_ group and -CH_2_- group at 2928 cm^− 1^ and 2857 cm^− 1^ indicated that the KH550 was successfully introduced on the surface of CF. The peak at 1107 cm^− 1^ ascribing to the antisymmetric vibration of the Si-O-Si group emerged and the peak at 801 cm^− 1^ attributed to the symmetrical stretching vibrations of the Si-O group emerged. Consequently, successful grafting of nano-SiO_2_ to the CF was preliminarily confirmed. From the results of XPS, the relative content of N and Si elements increased compared to that of unmodified CF, indicating that KH550 was successfully grafted on CF surface. The content of the O and Si elements in the CF-SiO_2_–2 spectrum also increased, indicating that nano-SiO_2_ with lots of -OH functional groups and KH560 was grafted on CF surface. From the results of the C1s peaks and the Si2p peaks obtained from the high-resolution XPS spectra by peak fitting, It could be noted that the Si-O-Si peak increased distinctly in the CF-SiO_2_ specimen, which was attributed to the nano-SiO_2_ on the surface. This indicated that nano-SiO_2_ was successfully grafted on the surface of CF.

From the results of SEM and EDS, for CF-SiO_2_–1 and CF-SiO_2_–2 specimens, there were homogeneous nano-SiO_2_ particles distributed on the surface of CF. But for the CF-SiO_2_–3 specimen, lots of agglomerates were observed on CF surface, which was ascribed that the content of nano-SiO_2_ particles was so much that some nano-SiO_2_ particles couldn’t be grafted on CF surface but began to aggregate together because silanol groups on their surface were easy to form hydrogen bonds [41]. The results could be proved that the nano-SiO_2_ was grafted on CF surface, and the distribution could be controlled by the amount of nano-SiO_2_.

Nano-SiO_2_ particles as a good nucleating agent of PLLA can promote the crystallization, and the distribution of nano-SiO_2_ may lead to different crystallization structures. The interfacial crystallization behavior was studied to evaluate the interfacial interaction between CF and PLLA. The interfacial crystallization behavior could be used to evaluate the interfacial interaction between CF and PLLA. The crystallization behaviors of the PLLA, PLLA/CF and PLLA/CF-SiO_2_ specimens at 135 °C after holding for 3, 6, 9 and 12 min were studied by POM to clarify the effect of SiO_2_ on interfacial crystallization. It was found that a transcrystalline structure formed at the interface between CF-SiO_2_–2 and PLLA, which might be attributed to the good nucleating capacity of nano-SiO_2_ [42–44]. Generally speaking, transcrystalline structure was formed by the compress each other of spherulites along CF surface and thus grew perpendicularly. The formation of transcrystalline structure induced by nano-SiO_2_ on CF surface constructed interfacial interaction between CF and PLLA, forming good interfacial adhesion. It should be noted that some spherulites also appeared on CF surface in the PLLA/CF-SiO_2_–1 specimen, but the content of nano-SiO_2_ grafted on CF was less, providing a low concentration of nucleation sites. As a result, the transcrystalline structure couldn’t be formed between PLLA and CF (Fig. 4 c). For the PLLA/CF-SiO_2_–3 specimen, the excess nano-SiO_2_ formed larger aggregates on CF surface, resulting in irregular nucleation sites, and the growth of spherulites became disorder. It could be found that the interfacial crystallization morphology could be controlled by the content of nano-SiO_2_ grafted on CF. The results of SEM was consistent with the results of POM (Figs. 5 b -b1), indicating that there was no interfacial interaction between CF and PLLA. But it could be observed that many branched crystalline morphologies emerged along CF in the PLLA/CF-SiO_2_–2 specimen. The reason for this difference between CF and CF-SiO_2_–2 might be that the nano-SiO_2_ with large surface energy induced the PLLA chains to fold on or around them, then the PLLA crystal nuclei formed and eventually grew into high order crystalline structures [45].

**Fig. 9:**
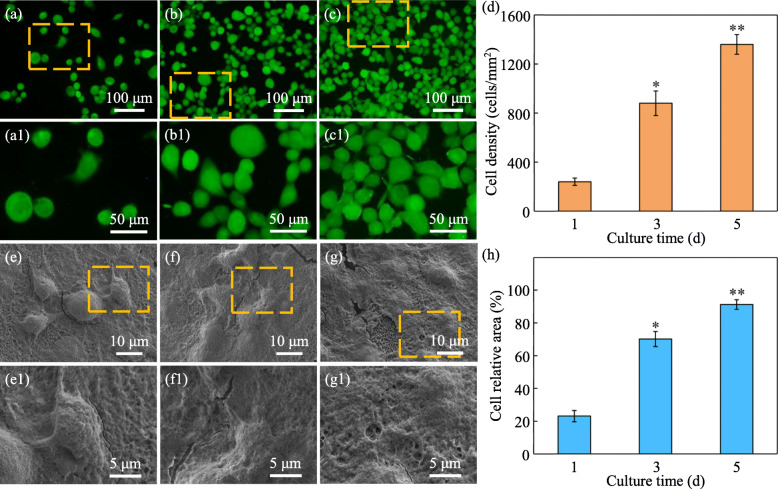
Schematic diagram depicting crystallization process of different specimens: (**a**) PLLA, (**b**) PLLA/CF and (**c**) PLLA/CF-SiO_2_

To better understand the crystallization process of PLLA, PLLA/CF and PLLA/CF-SiO_2_ specimens, a schematic is represented in Fig. 9. In the isothermal crystallization process, the ordered arrangement of PLLA molecular chains formed crystal nuclei firstly, then the crystal nuclei grew continually and formed crystal structure (Fig. 9 a). For the PLLA/CF specimen, since there was no interaction between PLLA and CF, most crystals still appeared randomly in PLLA matrix (Fig. 9 b). However, when the surface of CF was grafted with nano-SiO_2_, many crystal nuclei appeared on the surface of CF and gradually grew in a direction that was perpendicular to the long axis of CF, forming transcrystallinity structure along with the surface of CF (Fig. 9 c). Generally speaking, the slow crystallization rate and low crystallinity were mainly attributed to the poor ability of PLLA nucleation, whereas nano-SiO_2_ acted as a nucleating agent, reducing the nucleation barrier and shortening the nucleation period [46, 47]. When the nucleation density reached a certain point, the crystal growing in a parallel direction was hindered and then only grew in the direction that was perpendicular to the long axis of CF.

Bone scaffold should possess enough mechanical properties (Cortical bone: Tensile strength 50–151 MPa, Modulus 12–18 GPa. Cancellous bone: Tensile strength 1–5 MPa, Modulus 0.1–0.5 GPa [48]) to provide support for the growth and proliferation of cells [49, 50]. Tensile tests were carried out to explore the effect of CF grafted with nano-SiO_2_ on mechanical behavior. After adding CF, the enhancement of strength and modulus was achieved, which might be attributed to that the pulling out of CF from PLLA matrix consumed some fracture energy during the loading process. Whereas the bonding strength between them was not enough strong that the improvement in strength and modulus of the PLLA/CF composite was not obvious. Generally speaking, the mechanical properties of scaffold depend not only on the performance of fibers and polymer matrix but also on the interfacial property between them [51, 52]. Nano-SiO_2_ particles as good nucleating agent for PLLA were introduced onto the surface of CF, which might be beneficial to improve the interfacial bonding between them. For the CF-SiO_2_–1 specimen, there were fewer nano-SiO_2_ grafted on the CF surface, resulting in fewer nucleation sites and no obvious enhancement of mechanical properties. In the case of the CF-SiO_2_–3 specimen, the amount of SiO_2_ nanoparticles grafted on CF surface was too high that a mass of agglomerates formed on the CF surface. Some SiO_2_ nanoparticles agglomerated by the covalent bond formed among the silane coupling agent on their surface, but others agglomerated due to the huge specific surface area and extremely higher activity. Hence there would be some defects and weak interaction, which accounted for poor stress transfer between CF and PLLA matrix and declined in strength and modulus of scaffold. Among the three types of PLLA/CF-SiO_2_ specimens, the PLLA/CF-SiO_2_–2 possessed the best interfacial bonding strength, but that of the PLLA/CF-SiO_2_–1 and PLLA/CF-SiO_2_–3 were lower. When the amount of nano-SiO_2_ grafted on CF surface was less, the improvement of interfacial adhesion between them was not obvious. But for the PLLA/CF-SiO_2_–3, the agglomeration of nano-SiO_2_ on CF surface accounted for the local stress concentration and exhibited relatively poor tensile properties.

The high fiber strength and better interface between fiber and matrix are regarded as the main practical solutions to improve the mechanical properties of scaffold [53]. But the length of CF is shorter than the critical fiber length, resulting in that CF is not broken during the loading process, and therefore the interfacial debonding and fiber pulling-out act as the main mechanisms to enhance the mechanical properties by consuming the energy [54, 55]. To further study the interface bonding between CF-SiO_2_ and PLLA, SEM was applied to observe the fracture surface of the PLLA, PLLA/CF and PLLA/CF-SiO_2_ scaffold specimens after tensile tests. It could be found that strong interfacial bonding between CF-SiO_2_ and PLLA matrix was established because it could be observed that the matrix was fixed on the surface of CF from the SEM images of fracture surface. The reinforcement of interfacial properties might be attributed that the nano-SiO_2_ grafted on CF surface acting as a bridge increased the surface roughness and interface area and induced PLLA crystallization on CF surface. Besides, the formation of transcrystalline structure induced by nano-SiO_2_ grafted on CF surface imposed stronger mechanical locking effect on the CF/PLLA interface to further reinforce the interfacial bonding. A schematic diagram was used to explain the reinforcement mechanism between CF and matrix (Fig. 7 e). As for the PLLA/CF specimen, the surface of CF was smooth and there was no bonding or interaction between them, indicating that the interface was easily broken under the action of tensile force. However, for the PLLA/CF-SiO_2_ specimen, the nano-SiO_2_ particles with nucleation ability induced PLLA chains to fold on and around them, building strong adhesion between CF and PLLA. Besides, the nano-SiO_2_ particles grafted on CF surface constructed mechanical locking between them, which increased the resistance that CF was pulled out from the PLLA matrix. Even so, the interface was still broken when the nano-SiO_2_ particles debonded or the PLLA matrix was broken with the increase of tensile force.

An important criterion for bone scaffold is that it should possess the ability to support the growth and proliferation of cells [56–58]. As the PLLA/CF-SiO_2_–2 scaffold exhibited the best tensile properties, the PLLA/CF-SiO_2_–2 scaffold was selected to perform the assessment of cytocompatibility. It could be found that MG63 cells could grow and proliferate normally. It could be concluded that the introduction of nano-SiO_2_ and CF was nontoxic for MG63 cells. And with the extension of incubation time, MG63 cells grew and proliferated unceasingly on the PLLA/CF-SiO_2_–2 scaffold, indicating that the scaffold could provide a safe environment for the cells.

## Conclusion

In this study, nano-SiO_2_ particles grafted on the surface of CF by a self-assembly strategy were applied to improve the interfacial bonding between CF and PLLA in bone scaffold fabricated by SLS. In detail, CF with carboxyl groups and nano-SiO_2_ with hydroxyl groups were modified by KH550 and KH560, respectively. Then CF-KH550 and SiO_2_-KH560 were mixed, and nano-SiO_2_ particles were successfully anchored on the surface of CF via the combining of KH550 and KH560. The results of FTIR and XPS confirmed that nano-SiO_2_ was grafted on the surface of CF by the combination of KH550 and KH560. The introduction of nano-SiO_2_ improved the interfacial bonding by constructing transcrystallinity crystallization and strong mechanical locking between CF and PLLA. And the PLLA/CF-SiO_2_–2 scaffold had the maximum strength and modulus, which indicated that the uniform nano-SiO_2_ on CF surface effectively improved the interfacial bonding and enhanced the mechanical properties. In addition, MG63 cells could grow and proliferate well on the PLLA/CF-SiO_2_–2 scaffold. In short, the PLLA/CF-SiO_2_–2 scaffold with good mechanical properties and cytocompatibility had great potential for the application in bone tissue engineering.

## Data Availability

The datasets used and/or analysed during the current study are available from the corresponding author on reasonable request.

## Abbreviations

CF: Carbon fiber
PLLA: Poly (lactic acid)
KH560: 3-Glycidoxypropyltrimethoxysilane
KH550: 3-Aminopropyltriethoxysilane
SLS: Selective laser sintering
CF-COOH: CF with carboxyl groups
SiO_2_-OH: SiO_2_ nanoparticles with hydroxyl groups
PBS: Phosphate buffer saline
FTIR: Fourier transform infrared
XPS: X-ray photoelectron spectroscopy
SEM: Scanning electron microscope
POM: Polarized optical microscopy
EDS: Energy dispersive spectroscopy
DMEM: Dulbecco’s modified Eagle’s medium.
